# The Early Dead

**Published:** 1869-03

**Authors:** 


### THE EARLY DEAD.

It was stated at an anniversary of a New England college, that the average age of thirteen of its graduates who had died during the preceding year was less than thirty-three years. It is a melancholy reflection that so many young men die during their collegiate or early professional studies. That their death is properly chargeable to causes connected with the habits of college and seminary life is intimated in the fact, that of a class graduating in 1836, and numbering thirty-eight persons, only six had died during those twenty years, while six, an equal number, had died in that class during their collegiate course of four years; thus showing that the professional life of the mass of our clergymen is promotive of health. The reason is that they labor in small towns, and among a rural population requiring more or less visitation on foot and horseback, exercise amounting to hundreds of miles in a year, performed in all weathers, for appointments made which must be kept in spite of winds and storms and swollen streams.

Is not this a loud lesson to clergymen whose health is declining, that instead of wasting their time in doctoring they should first ascertain from a reliable physician what is really the matter, and if not contra-indicated, to get on a good trotting horse, and missionate among the mountain population in "the hill country" of our wide extended territory, and keep at it for a year or two, and let the congregations in whose service they have impaired their health see to it that they are liberally, adequately paid, as their proxies in sending the Gospel to the heathen of their own land, for there are many such among the mountains. When will the Church wake up to that most important subject—THE HYGIENE OF HER MINISTRY, and their liberal support.

